# Optic Nerve Infiltration in Retinoblastoma: Correlation of Histopathological and MRI Findings in Enucleated Eyes

**DOI:** 10.7759/cureus.100267

**Published:** 2025-12-28

**Authors:** Amna Ali, Saima Amin, Zeeshan Kamil, Ahmer Hamid, Muhammad Usama Idrees, Muhammad Tanweer Hassan Khan, Bhagwanti Kumari

**Affiliations:** 1 Ophthalmology, Layton Rahmatulla Benevolent Trust (LRBT) Tertiary and Teaching Eye Hospital, Karachi, PAK; 2 Ophthalmology, Layton Rahmatulla Benevolent Trust (LRBT), Karachi, PAK; 3 Ophthalmology/Orbit and Oculoplastics, Jinnah Medical and Dental College, Karachi, PAK; 4 Peadiatric Oncology, The Indus Hospital, Karachi, PAK; 5 General Ophthalmology, Layton Rahmatullah Benevolent Trust (LRBT) Free Tertiary Eye Care Hospital, Karachi, PAK

**Keywords:** diagnostic accuracy, enucleation, histopathology, magnetic resonance imaging (mri), optic nerve invasion, post-laminar optic nerve invasion (ploni), retinoblastoma

## Abstract

Background and objective

The accurate assessment of optic nerve invasion in retinoblastoma is critical for prognosis and treatment planning. Although MRI is routinely used for preoperative evaluation, it may fail to detect early microscopic invasion. Ensuring appropriate imaging interpretation and obtaining a sufficiently long optic nerve stump during enucleation are essential to avoid unnecessary morbidity related to adjuvant therapies. This study aimed to compare MRI findings with histopathological evaluation regarding optic nerve infiltration in enucleated eyes with group E retinoblastoma.

Materials and methods

This cross-sectional study included 45 patients with group E retinoblastoma who underwent upfront enucleation. Preoperative 1.5-T MRI was interpreted by an experienced neuroradiologist, and histopathology was performed according to the International Retinoblastoma Staging Working Group (IRSWG) guidelines. Sensitivity, specificity, positive predictive value (PPV), negative predictive value (NPV), and diagnostic accuracy of MRI for optic nerve invasion were calculated using histopathology as the reference standard.

Results

Histopathology detected post-laminar optic nerve invasion in 19 cases (42.2%), while MRI detected it in 17 cases (37.7%). Prelaminar invasion was identified in 12 eyes (26.7%) histologically, but was underestimated by MRI, which detected only five cases (11.1%). MRI showed a sensitivity of 76.0%, specificity of 72.7%, PPV of 80.9%, NPV of 66.7%, and overall accuracy of 75.0%.

Conclusions

MRI is a valuable noninvasive modality for assessing tumor extent and detecting post-laminar invasion; however, it consistently underestimates early (prelaminar) and microscopic optic nerve infiltration. Histopathology remains the gold standard for definitive evaluation and risk stratification. Regardless of MRI findings, obtaining an adequately long optic nerve stump during enucleation is crucial, as radiologic imaging may fail to detect microscopic invasion, and the cut-end status remains the most important prognostic determinant.

## Introduction

Retinoblastoma is the most common primary intraocular malignancy in children, originating from immature retinal cells [1]. It accounts for approximately 3% of all pediatric cancers and remains a significant cause of vision loss and mortality in children under five years of age [2]. The global incidence is estimated to be one case per 15,000-18,000 live births, corresponding to approximately 6,000 new cases annually [2]. More than 80% of cases occur in low- and middle-income countries, where delayed presentation and limited access to specialized care contribute to poorer outcomes. Although early detection and conservative therapies have advanced substantially, enucleation remains the treatment of choice for eyes with advanced intraocular disease [3].

According to the International Intraocular Retinoblastoma Classification (IIRC), group E retinoblastoma represents the most advanced stage of intraocular disease, often characterized by diffuse infiltrating tumor, total retinal detachment, neovascular glaucoma, anterior segment involvement, or dense vitreous seeding [4]. Given the substantial risk of extraocular extension and metastasis, primary enucleation is recommended for group E eyes to prevent disease spread and safeguard life [5]. Histopathological assessment following enucleation is the gold standard for identifying high-risk features, especially optic nerve and choroidal invasion [6]. These include prelaminar, laminar, post-laminar, and optic nerve resection margin involvement, as well as choroidal and scleral invasion, all of which inform the need for adjuvant therapy.

MRI is the imaging modality of choice for evaluating intraocular tumors and potential extraocular extension before enucleation [7]. Its superior soft-tissue contrast and multiplanar capability, combined with the absence of ionizing radiation, make it ideal for pediatric imaging [8]. MRI provides valuable information regarding optic nerve involvement, scleral breach, orbital extension, and intracranial disease [9]. However, despite advancements in high-resolution and contrast-enhanced imaging, MRI still demonstrates inconsistent correlation with histopathological findings. Reported sensitivity for detecting post-laminar optic nerve invasion ranges from 60 to 90%, with specificity between 70 and 95% [10,11]. False positives may arise from inflammation or vascular changes, whereas microscopic prelaminar invasion frequently goes undetected, leading to false negatives [12]. These limitations underscore the need for correlating MRI findings with histopathology.

Although several studies have endeavored to optimize MRI protocols and interpretation criteria [13,14], inconsistencies persist across institutions, and MRI often cannot reliably differentiate the precise levels of microscopic invasion. Histopathology, in contrast, allows definitive assessment of tumor extension at each optic nerve level, including the resection margin, which carries the strongest prognostic significance. Establishing a clear correlation between MRI and histopathology is therefore essential to determine MRI’s true predictive value in high-risk disease. Early recognition of optic nerve invasion on imaging may influence preoperative planning, including the need to obtain a sufficiently long optic nerve stump to keep the resection margin distant from tumor extension.

Accurate interpretation of invasion is equally important for risk stratification, enabling timely administration of adjuvant therapy and avoiding unnecessary treatment. Because treatment decisions ultimately rely on histopathology, overestimation on MRI cannot independently lead to overtreatment. Despite the widespread use of MRI, only a limited number of studies have directly correlated MRI findings with histopathological optic nerve invasion in group E retinoblastoma. With evolving imaging techniques and treatment strategies, validating MRI performance against histopathology remains vital for improving diagnostic accuracy, surgical planning, and overall patient management. This study aimed to determine the correlation between pre-enucleation MRI findings and histopathological levels of optic nerve infiltration in group E eyes, and to assess the accuracy of MRI in detecting prelaminar, laminar, and post-laminar invasion, with particular focus on resection margin involvement.

## Materials and methods

Study design, setting, and ethical approval

This retrospective observational correlation study aimed to determine the diagnostic accuracy of MRI in detecting optic nerve, scleral, and choroidal invasion in comparison with histopathology, the gold standard for diagnosis. The study focused exclusively on group E retinoblastoma eyes that underwent upfront enucleation without prior chemotherapy or focal therapy. The research was conducted in collaboration between the Departments of Radiology and Histopathology at Indus Hospitals, and all enucleations were performed at the Layton Rahmatulla Benevolent Trust (LRBT) over six months from April 1, 2025, to September 30, 2025. Ethical approval was obtained from the Institutional Review Committee of LRBT (approval no. LRBT/TTEH/ERC/3538/29; Date: 1 March 2025). Written informed consent was acquired from parents or legal guardians, and patient confidentiality was maintained throughout the study.

Study population

The sample size was calculated using OpenEpi version 3.01. Based on a 95% confidence interval (CI), 80% power, and an MRI sensitivity of 82% for detecting post-laminar optic nerve invasion [6], the minimum required sample size was 45 eyes. A non-probability purposive sampling technique was used to include all eligible group E eyes undergoing upfront enucleation during the study period. This sampling approach may introduce selection bias and may limit the generalizability of the results; therefore, it is acknowledged as a study limitation.

Eligibility and data collection

Inclusion criteria were eyes diagnosed as group E retinoblastoma according to the International Intraocular Retinoblastoma Classification (IIRC), undergoing upfront enucleation with complete preoperative MRI and full oncology workup, including cerebrospinal fluid cytology and bone marrow biopsy. Exclusion criteria included retinoblastoma groups A-D, any eyes receiving chemotherapy or focal therapy before enucleation, incomplete or technically inadequate MRI or histopathology slides, artifact-distorted optic nerve sections, patients evaluated with CT rather than MRI, extraocular retinoblastoma, and cases with intracranial extension. Demographic and clinical data were collected, including age, gender, laterality, family history, presenting features, and the duration from diagnosis to enucleation.

MRI technique

MRI examinations were performed using a 1.5-Tesla system with fat suppression and an eight-channel head coil. Scans were performed under general anesthesia when required. The MRI protocol consisted of axial T1-weighted, fat-suppressed T2-weighted, and pre- and post-contrast T1-weighted sequences in axial, coronal, and sagittal planes, along with high-resolution 3D FIESTA sequences for thin-section visualization of the optic nerve and sclera. Gadolinium contrast was administered at 0.1 mmol/kg. Diffusion-weighted imaging (DWI) was performed to evaluate diffusion restriction.

To enhance reproducibility, MRI technical parameters included a slice thickness of 1-3 mm, a field of view of 160-200 mm, a matrix of 256 × 256, and DWI performed using b-values of 0 and 1000 s/mm², along with high-resolution 3D FIESTA thin-section imaging for detailed optic nerve delineation. Whole-brain imaging was also obtained to exclude trilateral retinoblastoma. The median interval between MRI and enucleation was three days (range: one to seven days). All MRI scans were interpreted by an experienced radiologist blinded to histopathology. The optic nerve was evaluated for absence of invasion as well as prelaminar, laminar, post-laminar, and resection-margin invasion, as well as scleral and choroidal involvement.

Histopathology

Enucleated specimens were fixed in 100% buffered formalin and processed following the International Retinoblastoma Staging Working Group (IRSWG) protocol. Four blocks were prepared per eye: pupil-optic nerve section, two calottes, and the optic nerve resection margin. Sections were stained with hematoxylin and eosin and periodic acid-Schiff stains. Two independent pathologists evaluated tumor differentiation and the level of optic nerve, choroidal, and scleral invasion. Discrepancies between the two pathologists were resolved through a formal consensus review, and when necessary, joint correlation with the radiologist was performed to align imaging and histopathological findings and finalize the interpretation.

Statistical analysis

Data were entered into Microsoft Excel and analyzed using SPSS Statistics version 26 (IBM Corp., Armonk, NY). Continuous variables were expressed as mean ± standard deviation (SD), and normality was assessed with the Shapiro-Wilk test. Categorical variables were presented as frequencies and percentages. The diagnostic performance of MRI was evaluated by calculating sensitivity, specificity, positive predictive value (PPV), negative predictive value (NPV), and overall diagnostic accuracy, using histopathology as the reference standard. A p-value <0.05 was considered statistically significant.

## Results

A total of 45 enucleated eyes (n = 45) with group E retinoblastoma were included. The mean age at diagnosis was 3.8 ± 1.6 years. There were 27 males (60.0%) and 18 females (40.0%), yielding a male-to-female ratio of 1.5:1. The disease was unilateral in 39 patients (86.7%) and bilateral in six patients (13.3%). A positive family history was present in five patients (11.1%), while 40 (88.9%) had no known family history. Leukocoria was the most common presenting symptom (30 cases, 66.7%), followed by proptosis (11 cases, 24.4%) and strabismus (four cases, 8.9%). The mean interval between MRI and enucleation was 6.2 ± 3.8 days, helping minimize the likelihood of tumor progression influencing the imaging-histopathology correlation (Table 1).

**Table 1:** Demographic and clinical characteristics of the study participants (n = 45) SD: standard deviation; MRI: magnetic resonance imaging

Variable	Category	Values
Age at diagnosis, years, mean ± SD		3.8 ± 1.6
Gender, n (%)	Male	27 (60.0)
	Female	18 (40.0)
Laterality, n (%)	Unilateral	39 (86.7)
	Bilateral	6 (13.3)
Family history of retinoblastoma, n (%)	Present	5 (11.1)
	Absent	40 (88.9)
Mode of presentation, n (%)	Leukocoria	30 (66.7)
	Proptosis	11 (24.4)
	Strabismus	4 (8.9)
Duration between MRI and enucleation, days, mean ± SD		6.2 ± 3.8

Histopathological examination demonstrated a substantially higher frequency of prelaminar optic nerve invasion compared with MRI, confirming that MRI tended to underestimate early or microscopic infiltration. In contrast, MRI showed closer concordance with histopathology in detecting post-laminar optic nerve invasion, where detection rates were more comparable (n = 17; 37.7% vs. n = 19; 42.2%). A small subset of cases demonstrated no optic nerve invasion on either modality, indicating reasonable agreement in advanced disease but highlighting MRI’s limited sensitivity for subtle, prelaminar involvement (Table 2).

**Table 2:** Comparison of MRI and histopathology findings in optic nerve involvement (n = 45) MRI: magnetic resonance imaging

Level of optic nerve involvement	Histopathology, n (%)	MRI, n (%)
No invasion	8 (17.8)	10 (22.2)
Prelaminar invasion	12 (26.7)	5 (11.1)
Post-laminar invasion	19 (42.2%)	17 (37.7%)
Total	45 (100)	45 (100)

MRI demonstrated a moderate level of diagnostic accuracy in detecting optic nerve invasion when compared with histopathology as the gold standard. The results indicate that MRI performed reasonably well in identifying true cases of post-laminar involvement but showed some limitations in ruling out early or subtle infiltration. The agreement between MRI and histopathology was moderate, suggesting that while MRI is a useful noninvasive tool for preoperative assessment, it may not be sufficiently reliable for detecting microscopic or prelaminar invasion without histopathological confirmation (Table 3).

**Table 3:** Diagnostic performance of MRI in detecting optic nerve invasion (histopathology as the gold standard) MRI: magnetic resonance imaging; CI: confidence interval

Parameter	Value, %	95% CI
Sensitivity	76.0	61.8–90.2
Specificity	72.7	57.5–87.9
Positive predictive value (PPV)	80.9	66.5–95.3
Negative predictive value (NPV)	66.7	51.1–82.3
Overall accuracy	75.0	—
Cohen’s kappa agreement	0.52 (moderate agreement)	—

Histopathology identified scleral invasion in 14 cases (n = 14, 31.1%), while MRI detected it in only nine cases (n = 9, 20.0%). Choroidal invasion was found in 18 cases (n = 18, 40.0%) on histopathology and in 11 cases (n = 11, 24.4%) on MRI. Combined sclero-choroidal invasion was present in 10 cases (n = 10, 22.2%) on histopathology and six cases (n = 6, 13.3%) on MRI. The diagnostic accuracy of MRI was 77.8% for scleral invasion, 73.3% for choroidal invasion, and 76.0% for combined involvement (Table 4).

**Table 4:** Comparison of MRI and histopathology in scleral and choroidal involvement MRI underestimates scleral and choroidal infiltration compared to histopathology, particularly in cases with microscopic extraocular extension MRI: magnetic resonance imaging

Parameter	Histopathology positive, n (%)	MRI positive, n (%)	Sensitivity, %	Specificity, %	Accuracy, %
Scleral invasion	14 (31.1%)	9 (20.0%)	64.3%	86.2%	77.8%
Choroidal invasion	18 (40.0%)	11 (24.4%)	61.1%	83.3%	73.3%
Combined sclero-choroidal invasion	10 (22.2%)	6 (13.3%)	60.0%	87.9%	76.0%

In some cases where CT scans were performed due to the unavailability of MRI in remote areas, CT demonstrated limited diagnostic performance in all parameters except for detecting intraocular calcifications. MRI showed notably higher sensitivity and specificity than CT for identifying optic nerve, scleral, and choroidal involvement, with CT particularly limited in differentiating soft-tissue details. These findings reinforce the superior diagnostic role of MRI over CT for evaluating local tumor extension and optic nerve infiltration in retinoblastoma, although MRI still underestimates early microscopic or subtle prelaminar, scleral, and choroidal invasion (Table 5).

**Table 5:** Diagnostic comparison between MRI and CT scan in optic nerve and extraocular involvement MRI provides better tissue detail and soft-tissue differentiation than a CT scan, though it still lacks sufficient sensitivity for early prelaminar and subtle scleral or choroidal invasion. CT scan is markedly inferior in all parameters MRI: magnetic resonance imaging; CT: computed tomography

Site of involvement	Sensitivity %, MRI	Specificity %, MRI	Sensitivity %, CT	Specificity %, CT
Optic nerve invasion	78.1	70.0	48.3	65.0
Scleral invasion	64.3	86.2	40.0	68.9
Choroidal invasion	61.1	83.3	37.8	70.2
Extraocular extension	72.0	88.0	45.0	80.0

## Discussion

In this study, MRI demonstrated moderate overall accuracy for detecting optic nerve invasion but consistently underdetected prelaminar and laminar involvement that histopathology identified, while correctly detecting most cases of post-laminar disease. Clinically, this underestimation has important implications: in our cohort, 12 eyes (26.7%) with prelaminar invasion and zero eyes with laminar invasion would have been underestimated if relying solely on MRI, highlighting the risk of incomplete surgical planning or delayed adjuvant therapy. These findings align with contemporary literature reporting higher MRI sensitivity for macroscopic or post-laminar invasion but lower sensitivity for subtle prelaminar disease, with pooled analyses estimating a sensitivity of approximately 61% and specificity of 88% for post-laminar optic nerve invasion [10].

Recent studies, including those by De Bloeme et al., emphasized that high-spatial-resolution MRI markers such as optic nerve thickening and specific enhancement patterns can improve detection of early post-laminar invasion, yet microscopic invasion remains difficult to detect, necessitating histopathological confirmation [11]. Our findings reinforce this pattern, demonstrating that MRI reliably identifies post-laminar involvement but underestimates early disease. To mitigate the risk of leaving residual tumor, it is imperative to take a long optic nerve stump (10-15 mm) even when MRI shows no involvement, integrating this recommendation directly with the observed false negatives for prelaminar or laminar invasion [15]. A retinoblastoma clinical practice review states that surgeons should aim for a long optic nerve stump (>10 mm) to help ensure adequate margin clearance and reliable pathological evaluation [16].

Other reviews and imaging guideline updates have underlined the same diagnostic tradeoffs and the practical rationale for performing MRI preoperatively despite its limits. A recent imaging review summarized that MRI provides essential staging information, detects intracranial disease, and helps surgical planning, but its NPV for microscopic optic nerve head invasion remains limited; therefore, histopathology remains the reference standard for final risk stratification. Our finding of a modest NPV therefore aligns with contemporary guidance, which positions MRI as a complementary tool rather than a substitute for histopathological evaluation [17,18].

Regarding scleral and choroidal invasion, MRI sensitivity was reduced for subtle or microscopic involvement, consistent with prior reports. In our study, histopathology detected 14 cases (31.1%) of scleral invasion and 18 cases (40.0%) of choroidal invasion, whereas MRI identified only nine (20.0%) and 11 (24.4%) cases, respectively. While MRI specificity remained high, the underdetection of early or microscopic invasion underscores the importance of histopathology for definitive risk stratification and decisions regarding adjuvant therapy [10,19,20]. Comparing MRI with CT, our data confirm that MRI provides superior soft-tissue contrast and multiplanar resolution, resulting in better detection of optic nerve, scleral, and choroidal involvement. CT remains limited to identifying gross extension and calcifications and is markedly inferior for detailed preoperative staging, supporting current guidelines that reserve CT for selected indications [21,22].

Potential technical improvements, such as moving from 1.5 Tesla to 2-3 Tesla MRI, may incrementally increase spatial resolution and signal-to-noise ratio, theoretically improving sensitivity for prelaminar and laminar invasion; published data suggest that higher field strength and thinner slices can increase detection of early optic nerve infiltration, though microscopic foci may still remain below the resolution threshold [22,23]. Standardization of imaging protocols, including high-resolution sequences, dedicated orbit coils, and diffusion metrics, could further reduce interobserver variability and enhance diagnostic reliability.

This study is limited by its single-center, retrospective design and modest sample size, which may limit generalizability. By excluding post-chemotherapy enucleations, we ensured a cleaner MRI-pathology correlation but narrowed applicability to eyes treated with primary globe-sparing therapy. While consensus review was used to resolve discrepancies between radiologists and pathologists, some degree of subjective interpretation persists. Future directions include prospective multicenter studies with standardized MRI protocols, blinded central reads, quantitative imaging markers, and predictive modeling to improve preoperative detection of early or microscopic invasion and reduce variability between centers.

MRI provides valuable preoperative information and detects most cases of post-laminar optic nerve invasion, but it systematically underestimates prelaminar, laminar, and microscopic scleral or choroidal disease. Clinicians should use MRI as a complementary tool to guide surgical planning, including taking a 10-15 mm optic nerve stump, while histopathology remains the gold standard for definitive risk assessment and adjuvant therapy decisions. Ongoing technical refinements, higher-field imaging, and collaborative multicenter validation are needed to narrow the gap between radiologic suspicion and microscopic confirmation, ultimately improving clinical outcomes for patients with advanced retinoblastoma.

## Conclusions

MRI remains the preferred imaging modality for preoperative assessment and surgical planning in retinoblastoma; however, it has limited sensitivity in detecting early tumor invasion, particularly at the prelaminar and laminar optic nerve levels. Histopathology, in contrast, unequivocally confirms prelaminar, laminar, post-laminar, scleral, and choroidal invasion, as well as involvement of the optic nerve resection margin. These findings reaffirm that histopathology remains the gold standard for identifying microscopic tumor extension and defining high-risk features. MRI’s strength lies in noninvasively delineating gross tumor extent and identifying post-laminar involvement, which can guide timely management and reduce the risk of overt extraocular spread. Advances in imaging, such as high-resolution sequences and quantitative radiologic markers, may improve the detection of subtle or microscopic invasion and refine surgical and adjuvant therapy decisions. Clinically, these limitations mean that even when MRI shows no post-laminar invasion, surgeons should aim to resect the maximum safe length of the optic nerve, recognizing anatomical constraints, because a negative optic nerve margin remains the most critical prognostic factor. Ultimately, combining precise radiologic assessment with histopathologic confirmation enables optimal risk stratification, guides adjuvant therapy, and may contribute to improved patient outcomes and survival in children with advanced retinoblastoma.

## References

[1] Kumari A, Singh SP, Kumar P (2025). A comprehensive review of the epidemiology, pathophysiology, risk factors, and treatment strategies for retinoblastoma. Diseases.

[2] Feng Y, Feng X, Lv Y (2024). Worldwide burden of retinoblastoma from 1990 to 2021. Ophthalmic Res.

[3] Zhu H, Zhao M, Zheng J (2025). Global, regional, and national burden of retinoblastoma in children aged under 10 years from 1990 to 2021 and projections for future disease burden. Sci Rep.

[4] Sedaghat A, Raval V, Jia R, Fan X, Singh AD (2024). Advanced intraocular retinoblastoma: evolving criteria for group E disease. Can J Ophthalmol.

[5] Wang N, Liu R, Li J (2022). Long-term efficacy of enucleation combined with primary orbital implantation in children with retinoblastoma histopathological invasion of optic nerve. Front Neurol.

[6] Abusayf MM, Alkatan HM, Elkhamary S, Almesfer SA, Maktabi AM (2020). Histopathological assessment of optic nerve invasion guided by radiological findings in enucleated globes with retinoblastoma. BMC Ophthalmol.

[7] Niendorf T, Beenakker JM, Langner S (2021). Ophthalmic magnetic resonance imaging: where are we (heading to)?. Curr Eye Res.

[8] Gallo-Bernal S, Bedoya MA, Gee MS, Jaimes C (2023). Pediatric magnetic resonance imaging: faster is better. Pediatr Radiol.

[9] Nagesh CP, Rao R, Hiremath SB, Honavar SG (2021). Magnetic resonance imaging of the orbit, part 2: characterization of orbital pathologies. Indian J Ophthalmol.

[10] Cho SJ, Kim JH, Baik SH, Sunwoo L, Bae YJ, Choi BS (2021). Diagnostic performance of MRI of post-laminar optic nerve invasion detection in retinoblastoma: a systematic review and meta-analysis. Neuroradiology.

[11] de Bloeme CM, Jansen RW, Göricke S (2024). Optic nerve thickening on high-spatial-resolution MRI predicts early-stage postlaminar optic nerve invasion in retinoblastoma. Eur Radiol.

[12] Wiwatwongwana D, Kulniwatcharoen P, Mahanupab P, Visrutaratna P, Wiwatwongwana A (2021). Accuracy of computed tomography and magnetic resonance imaging for detection of pathologic risk factors in patients diagnosed with retinoblastoma. Curr Eye Res.

[13] Chow LS, Paley MN (2021). Recent advances on optic nerve magnetic resonance imaging and post-processing. Magn Reson Imaging.

[14] O'Shaughnessy E, Senicourt L, Mambour N, Savatovsky J, Duron L, Lecler A (2024). Toward precision diagnosis: machine learning in identifying malignant orbital tumors with multiparametric 3 T MRI. Invest Radiol.

[15] All India Ophthalmological Society (2025). All India Ophthalmological Society: AIOS Surgical Guidelines. All India Ophthalmological Society.

[16] Nag A, Khetan V (2024). Retinoblastoma-a comprehensive review, update and recent advances. Indian J Ophthalmol.

[17] Pai V, Muthusami P, Ertl-Wagner B (2024). Diagnostic imaging for retinoblastoma cancer staging: guide for providing essential insights for ophthalmologists and oncologists. Radiographics.

[18] Cardoen L, Sirin S, Galluzzi P (2025). Correction: Practical guidelines on imaging of retinoblastoma: a 2025 update on behalf of the European Retinoblastoma Imaging Collaboration and the European Retinoblastoma Group. Eur Radiol.

[19] Zhou M, Tang J, Fan J (2024). Recent progress in retinoblastoma: pathogenesis, presentation, diagnosis and management. Asia Pac J Ophthalmol (Phila).

[20] Onishi T, Nishina S, Yokoi T (2024). Outcomes of five cases of retinoblastoma with optic nerve invasion on imaging. Jpn J Ophthalmol.

[21] Schaiquevich P, Francis JH, Cancela MB, Carcaboso AM, Chantada GL, Abramson DH (2022). Treatment of retinoblastoma: what is the latest and what is the future. Front Oncol.

[22] Jansen RW, van der Heide S, Cardoen L (2022). Magnetic resonance imaging can reliably differentiate optic nerve inflammation from tumor invasion in retinoblastoma with orbital cellulitis. Ophthalmology.

[23] Zhao J, Cui R, Li L, Zhao B, Chen L (2024). Multimodal imaging for the differential diagnosis and efficacy evaluation of intraocular retinoblastoma in children with selective ophthalmic artery infusion. Transl Pediatr.

